# Estimands in cluster-randomized trials: choosing analyses that answer the right question

**DOI:** 10.1093/ije/dyac131

**Published:** 2022-07-14

**Authors:** Brennan C Kahan, Fan Li, Andrew J Copas, Michael O Harhay

**Affiliations:** MRC Clinical Trials Unit at UCL, Institute of Clinical Trials and Methodology, London, UK; Department of Biostatistics, Yale University School of Public Health, New Haven, CT, USA; Center for Methods in Implementation and Prevention Science, Yale University School of Public Health, New Haven, CT, USA; MRC Clinical Trials Unit at UCL, Institute of Clinical Trials and Methodology, London, UK; Clinical Trials Methods and Outcomes Lab, PAIR (Palliative and Advanced Illness Research) Center, Perelman School of Medicine, University of Pennsylvania, Philadelphia, PA, USA; Department of Biostatistics, Epidemiology, and Informatics, Perelman School of Medicine, University of Pennsylvania, Philadelphia, PA, USA

**Keywords:** Participant-average treatment effect, cluster-average treatment effect, cluster-randomized trial, estimands, informative cluster size, independence estimating equations, estimands

## Abstract

**Background:**

Cluster-randomized trials (CRTs) involve randomizing groups of individuals (e.g. hospitals, schools or villages) to different interventions. Various approaches exist for analysing CRTs but there has been little discussion around the treatment effects (estimands) targeted by each.

**Methods:**

We describe the different estimands that can be addressed through CRTs and demonstrate how choices between different analytic approaches can impact the interpretation of results by fundamentally changing the question being asked, or, equivalently, the target estimand.

**Results:**

CRTs can address either the participant-average treatment effect (the average treatment effect across participants) or the cluster-average treatment effect (the average treatment effect across clusters). These two estimands can differ when participant outcomes or the treatment effect depends on the cluster size (referred to as ‘informative cluster size’), which can occur for reasons such as differences in staffing levels or types of participants between small and large clusters. Furthermore, common estimators, such as mixed-effects models or generalized estimating equations with an exchangeable working correlation structure, can produce biased estimates for both the participant-average and cluster-average treatment effects when cluster size is informative. We describe alternative estimators (independence estimating equations and cluster-level analyses) that are unbiased for CRTs even when informative cluster size is present.

**Conclusion:**

We conclude that careful specification of the estimand at the outset can ensure that the study question being addressed is clear and relevant, and, in turn, that the selected estimator provides an unbiased estimate of the desired quantity.

Key Messages

- Decisions about how to analyse cluster-randomized trials can unintentionally result in answering different questions about interventions (i.e. target estimands). The ‘participant-average treatment effect’ answers the question ‘How effective is the intervention for the average participant?’ whereas the ‘cluster-average treatment effect’ answers the question ‘How effective is the intervention for the average cluster?’.
- The answers to these two questions can differ when outcomes or the effect of the intervention depends on the cluster size (referred to as ‘informative cluster size’), which can occur for several reasons, including inherent differences between larger and small clusters (e.g. experience, number of staff or quality of care) or systematic differences between participants in smaller or larger clusters (e.g. socio-economic status). This issue applies to both collapsible (e.g. difference in means) and non-collapsible (e.g. odds ratio) effect measures.
- The key difference between the two estimands is in how they are weighted. The participant-average treatment effect gives equal weight to each participant and the cluster-average treatment effect gives equal weight to each cluster. Therefore, an estimator that gives equal weight to each participant (e.g. an analysis of unweighted individual-level data or an analysis of cluster-level summaries weighted by the cluster size) will target the participant-average treatment effect and an estimator that gives equal weight to each cluster (e.g. an analysis of unweighted cluster-level summaries or an analysis on individual-level data weighted by the inverse of the cluster size) will target the cluster-average treatment effect.
- However, standard estimators, such as mixed-effects models or generalized estimating equations with an exchangeable correlation structure, are likely to produce biased estimates for both the participant-average and cluster-average treatment effects when the cluster size is informative. Conversely, independence estimating equations and approaches based on cluster-level summaries are unbiased when informative cluster sizes are present.
- Specifying the treatment effect of interest (the estimand) is essential to ensure that the study question being addressed is clear and that the appropriate statistical method is chosen to estimate the desired effect.

## Background

Cluster-randomized trials (CRTs) involve randomizing clusters of participants (such as hospitals, schools or villages) to different interventions.^1^^,^^2^ CRTs are typically used when the intervention is targeted at the cluster level or when there is a risk of contamination between treatment groups.

The analysis of CRTs must account for correlation between participants from the same cluster in order to obtain valid standard errors;^1–3^ however, different methods of doing so can alter the interpretation of the treatment effect being estimated and thus answer a different question to the one intended.^4–13^ Herein, we describe how CRTs can estimate different treatment effects, or ‘estimands’ (a precise description of the treatment effect to be estimated,^14^ see Box 1), and how to choose an estimator that aligns with the study question.

Box 1 Estimands, estimators, and estimates in cluster-randomized trials

**Estimand:** a precise description of the treatment effect investigators aim to estimate from the trial. An estimand comprises five aspects: (i) population; (ii) treatment conditions; (iii) endpoint; (iv) summary measure (e.g. difference in means, risk ratio, etc.); and (v) how intercurrent events are to be handled. In cluster-randomized trials it is important to define whether the estimand will reflect a ‘participant-average treatment effect’ or a ‘cluster-average treatment effect’ (see Table 2)
**Estimator:** The method of analysis used to calculate the estimated treatment effect. Valid estimators for the ‘participant-average’ and ‘cluster-average treatment effects’ are shown in Table 2
**Estimate:** the numerical value computed by the estimator [e.g. in a cluster-randomized trial that reported a mean difference of 3.0 (95% CI 2.5 to 3.5), the value 3.0 represents the ‘estimate’]

## Participant-average and cluster-average treatment effects

To see how different estimators can address different questions, consider the hypothetical data in Table 1. In this fictional CRT, there are three small clusters (10 participants each) and three large clusters (100 participants each) and the true treatment effect (based on a difference in potential outcomes under intervention vs control) is 5 in the small clusters and 1 in the large clusters.

**Table 1:** Data from a fictional cluster-randomized trial

Cluster (i)	Number of participants ($n_{i}$)	True treatment effect in cluster i
1	10	5
2	10	5
3	10	5
4	100	1
5	100	1
6	100	1

i denotes cluster number and $n_{i}$ denotes the number of participants in cluster i.

If we calculate the treatment effect as an average across all individuals in the trial, we obtain:

$$\frac{\left(10\right)\left(5\right)+\left(10\right)\left(5\right)+\left(10\right)\left(5\right)+\left(100\right)\left(1\right)+\left(100\right)\left(1\right)+\left(100\right)\left(1\right)}{10+10+10+100+100+100}=1.4$$

which is an average of the treatment effects, weighted by the number of participants they correspond to.

However, if we calculate the treatment effect as an average across clusters, we obtain:

$$\frac{5+5+5+1+1+1}{6}=3$$

Hence, these two approaches lead to two different treatment effects and thus interpretations around the usefulness of the intervention (Table 2): (i) the average treatment effect across participants (participant-average treatment effect); and (ii) the average treatment effect across clusters (cluster-average treatment effect). As we can see from the results above, these two treatment effects can differ substantially. In this example, the cluster-average treatment effect is more than twice the size of the participant-average treatment effect.

**Table 2:** Estimands and estimators for cluster-randomized trials

Estimand	Description	Method of estimation
Participant-average treatment effect^a^	Average treatment effect across participants (i.e. ‘How effective is the intervention for the average participant?’). Here, each participant is given equal weight	**Cluster-level analysis** Calculate cluster-level summaries (e.g. mean outcome in each cluster) Analyse cluster-level summaries using a weighted regression model (with weights equal to $n_{i}$, and robust standard errors) to give each participant equal weight. **Participant-level analysis** Independence estimating equations on participant-level data (which give equal weight to each participant) using robust standard errors that account for correlation between participants in the same cluster (e.g. GEE with a working independence correlation structure and robust standard errors or maximum-likelihood/least squares estimators with cluster-robust standard errors)
Cluster-average treatment effect^a^	Average treatment effect across clusters (i.e. ‘How effective is the intervention for the average cluster?’). Here, each cluster is given equal weight	**Cluster-level analysis** Calculate cluster-level summaries (e.g. mean outcome in each cluster) Analyse cluster-level summaries using regression model (unweighted, so that each cluster is given equal weight) **Participant-level analysis** Weighted independence estimating equations on participant-level data using robust standard errors, with inverse cluster-size weights equal to $\frac{1}{n_{i}}$ to give equal weight to each cluster

a For collapsible effect measures (e.g. the difference in means, risk difference or risk ratio), the participant-average and cluster-average estimands will coincide unless the treatment effect varies according to cluster size. For non-collapsible effect measures (e.g. odds ratio, hazard ratio), the participant-average and cluster-average estimands will only coincide if there is no difference in either outcomes or treatment effects between small and large clusters. GEE, generalized estimating equation.

## When cluster-average and participant-average treatment effects will differ

Whether the cluster-average and participant-average treatment effects differ will depend on whether there is ‘informative cluster size’, which denotes an association between cluster size (i.e. the number of participants in each cluster) and the outcomes in that cluster.^6^^,^^7^^,^^9–13^

There are two types of informative cluster size: (i) outcomes differ between small and large clusters but the treatment effect is the same (e.g. the baseline event rate is 10% in small clusters and 20% in large clusters but the odds ratio in both is 0.75); and (ii) the treatment effect differs between small and large clusters, i.e. the cluster size interacts with the treatment arm, or in other words, modifies the treatment effect (e.g. the odds ratio is 0.75 in small clusters and 0.50 in large clusters).

Informative cluster size can occur for several reasons, such as differences in staff experience or levels of care between larger and smaller hospitals, or differences in socio-economic status between larger urban schools compared with smaller rural schools. Informative cluster size then arises if these factors that differ between small and large clusters also affect the outcome or interact with the treatment group. Conversely, informative cluster size can also occur as an artefact of the trial (e.g. if better-performing clusters not only achieve better participant outcomes or treatment effects but are also more adept at recruiting participants to the study and hence have larger sample sizes than worse-performing clusters).

For collapsible treatment effect measures^15^ (such as a difference in means, risk difference or risk ratio), the participant-average and cluster-average treatment effects will coincide unless the second type of informative cluster size occurs, in which the treatment effect depends on the number of enrolled participants in a cluster. When this occurs, the value of the two estimands will differ (as is the case in Table 1). Importantly, for collapsible effect measures, these two estimands will coincide even if the first type of informative cluster size occurs (where outcomes depend on cluster size).

However, for non-collapsible effect measures (such as an odds ratio or hazard ratio), the participant-average and cluster-average treatment effects will only coincide if neither type of informative cluster size occurs (i.e. there is no difference in either outcomes or treatment effects between small and large clusters); if either type occurs, then the value of the two estimands will differ.^6^^,^^7^^,^^11^^,^^12^^,^^16^

In general, the larger the variation in cluster size, the larger the difference will be between the participant-average and cluster-average treatment effects when cluster size is informative.

## Specifying the estimand

Given that the participant-average and cluster-average treatment effects address fundamentally different questions, it is important to be clear on which question the trial is designed to answer and to choose the estimand that is most relevant for a specific trial objective. This will, of course, depend on the specific aims of the trial. For instance, if hospitals act as the cluster and the outcome relates to individual participants (e.g. a hospital-level intervention aiming to reduce mortality in presenting patients), then the participant-average treatment effect will be of most interest, as this represents the population impact of switching from the control to intervention. However, in a trial aiming to reduce unnecessary prescribing of antibiotics, in which doctors act as the cluster and outcomes are measured on each participant they treat, then a cluster-average treatment effect may also be of interest, as this provides the intervention’s effect on the clinician’s prescribing habits.

It is also important to specify other aspects that make up the estimand,^14^ including the (i) population of interest; (ii) treatment conditions; (iii) endpoint; (iv) population-level summary measure (how outcomes under different treatment conditions are to be compared, e.g. the difference in means); and (v) handling of intercurrent events, such as treatment discontinuation or switching. Further information on the specification of these aspects is available elsewhere.^8^^,^^17–23^ In addition to whether the cluster-average vs participant-average treatment effect is of interest, proper specification of these aspects will help to ensure that the study question is both clear and relevant, and that statistical methods estimate the quantity of interest.

## Methods of analysing cluster-randomized trials

The key difference between the two estimands is how participant outcomes are weighted (the participant-average treatment effect gives equal weight to each participant and the cluster-average treatment effect gives equal weight to each cluster). Hence, which estimand a particular estimator will correspond to depends on how the estimator weights the data: if it provides equal weight to each participant, it will target the participant-average treatment effect, whereas if it gives equal weight to each cluster, it will target the cluster-average treatment effect.

In CRTs, treatment effects can be estimated either by implementing an analysis either at the cluster level or the individual level.^1–3^^,^^24^ A cluster-level analysis involves calculating a summary measure for each cluster (e.g. the mean outcome across participants in that cluster) and then comparing cluster-level summaries. In contrast, an individual-level analysis typically involves analysing participant-level outcomes using a regression model that accounts for correlations between participants from the same cluster.

If implemented in the manner described above, a cluster-level analysis will target a cluster-average treatment effect, whereas an individual-level analysis will target the participant-average treatment effect. However, we can reweight a cluster-level analysis to give each participant equal weight to target a participant-average treatment effect. Similarly, we could reweight individual-level analyses to give equal weight to each cluster to target a cluster-average treatment effect. For a cluster-level analysis, this is done by weighting each cluster by the number of participants within that cluster, and for a participant-level analysis, this is done by weighting each individual by the inverse number of participants in that cluster (Table 2 provides a summary of these approaches).

## How statistical decisions can lead to different estimands

Often in CRTs, the choice of the estimator used for analysis is based on statistical considerations.^24^^,^^25^ Individual-level analyses are often more flexible (e.g. they are easier to adapt to allow for additional levels of clustering or to adjust for baseline covariates). However, individual-level analyses can also underestimate standard errors when the number of clusters is small.^3^^,^^24–28^ Although there are several small-sample adjustment methods that improve standard error estimation in such settings,^29–31^ cluster-level analyses are sometimes preferred as they are less prone to small-sample biases, as shown in simulation studies.^24^

However, as described above, these technical decisions can result in the targeting of different treatment effects, and so by changing the analysis for statistical reasons, study investigators may inadvertently be answering a fundamentally different question to the one they intended. Hence, when choosing an analysis method, it is important not only to ensure statistical validity but also to ensure it targets the desired estimand.

## Bias from common estimators for the participant-average treatment effect

Another issue in CRTs is that certain commonly used estimators can be biased when the cluster size is informative.^6^^,^^7^^,^^9–13^ This is the case for two individual-level analyses frequently used to estimate the participant-average treatment effect: mixed-effects models with a random intercept for cluster and generalized estimating equations (GEEs) with an exchangeable working correlation structure.^32^

**Figure 1:**
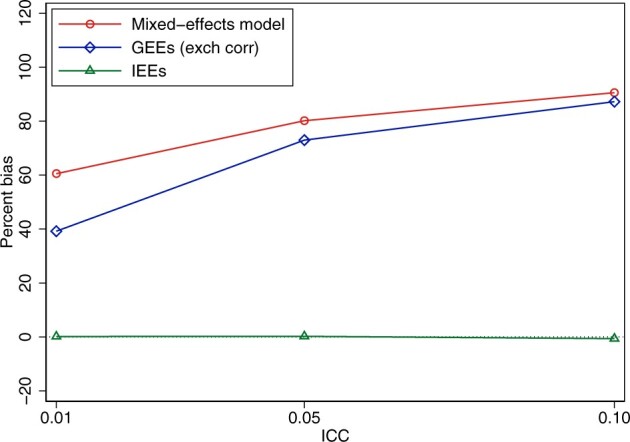
Bias from common estimators in cluster-randomized trials for the participant-average treatment effect when there is informative cluster size. Data were simulated based on the fictional trial given in Table 2. In each simulated data set, there were 60 clusters (30 with *n* = 10, 30 with *n* = 100). We generated a continuous outcome using true treatment effects of 5 in small clusters and 1 in large clusters [the residual standard deviation was set to 5 and the between-cluster standard deviation was calculated based on the desired intraclass correlation coefficient (ICC)]. We analysed data using (i) a mixed-effects model with random intercepts; (ii) generalized estimating equations (GEEs) with an exchangeable working correlation structure; and (iii) independence estimating equations (IEEs), implemented using a linear regression model with cluster-robust standard errors (note that weighted cluster-level analyses provide identical estimates to IEEs so will have identical bias). We used 2000 replications for each scenario. Bias was calculated as the difference between the average of the estimates for a particular estimator and the true value of the treatment effect (1.36). The ICC (on the *x*-axis) denotes the intraclass correlation coefficient (a measure of the correlation between participants in the same cluster). Statistical code is available in the Supplementary Material, available as Supplementary data at *IJE* online.

The reason these two estimators can be biased is that they do not give equal weight to each participant. Instead, clusters are weighted by their inverse-variance, which is a function of both the cluster size and the intraclass correlation coefficient (the degree of correlation between participants in the same cluster).^33^^,^^34^ Hence, each participant's weight in the analysis depends on which cluster they belong to and some participants will be given more weight than others. Thus, the weighting from mixed-effects models or GEEs with an exchangeable working correlation structure does not correspond to the participant-average treatment effect (which requires equal weight for each participant), resulting in biased effect estimates when there is informative cluster size.^6^^,^^7^^,^^9–12^^,^^26^^,^^33^ The emergence of bias due to informative cluster size can be seen in Figure 1, which illustrates the bias for these estimators in our previously described fictional example.

Of note, these estimators will be biased for collapsible effect measures (difference in means, risk difference, risk ratio) if the second type of informative cluster size occurs (where the treatment effect differs between smaller and larger clusters); however, they will be biased for non-collapsible effect measures (odds ratio, hazard ratio) if either type of informative cluster size occurs (either the outcome or treatment effect differs between smaller and larger clusters). Further, it is important to note that this bias occurs even with large sample sizes (i.e. it is not a small-sample bias issue). As an intuitive explanation, although GEEs are typically robust to misspecifications of the working correlation structure under large sample sizes, this requires the mean model to be correctly specified, which is not the case under informative cluster size. Finally, although we have discussed bias in these estimators in relation to the participant-average treatment effect, we note they will be biased for the cluster-average treatment effect as well because the weighting scheme from these estimators also differs from the weighting used in defining the cluster-average estimand.

Importantly, the bias produced from mixed-effects models and GEEs with an exchangeable working correlation structure arises when the cluster size is informative. In the absence of informative cluster size, these estimators will be unbiased for the participant-average treatment effect.

## Unbiased estimators for the participant-average treatment effect

Under informative cluster size, independence estimating equations (IEEs) are typically required to obtain unbiased estimates of the participant-average treatment effect.^6^^,^^7^^,^^9–13^^,^^35^ IEEs employ an independence working correlation structure in conjunction with robust standard errors to account for clustering.^36^ They can be easily implemented in standard software such as Stata, SAS or R by using GEEs with a working independence assumption and robust standard errors or by using a standard regression model estimated by maximum likelihood/least squares with cluster-robust standard errors. Alternatively, weighted cluster-level analyses can also provide unbiased estimates (Table 2).

We provide example Stata and R code in Table 3 denoting how to implement the different estimators. However, IEEs can be less efficient than mixed-effects models or GEEs with an exchangeable working correlation structure so the latter could be used if there is a strong reason a priori to believe that the cluster size will not be informative.

**Table 3:** Example Stata and R code to implement different estimators

Estimand	Estimator	Stata code	R code
Participant-average treatment effect	Participant-level (unweighted independence estimating equations)	xtset centre xtgee y treat, family(normal) link(identity) corr(ind) vce(robust)	library(geepack) geeglm(y ∼ treat, id=centre, family=gaussian(‘identity’), corstr=‘independence’)
	Cluster-level (weighted)^a^	collapse (count) n = y (mean) y treat, by(centre) reg y treat [pweight = n]	library(sandwich) ybar <- as.numeric(tapply(y, centre, mean)) treatcl <- as.numeric(tapply(treat, centre, mean)) model <- lm(ybar ∼ treatcl, weights=n) vcovHC(model, type=‘HC0’)
Cluster-average treatment effect	Participant-level (weighted independence estimating equations)	xtset centre xtgee y treat [pweight = 1/n], family(normal) link(identity) corr(ind) vce(robust)	library(geepack) nrep <- rep(n, n) geeglm(y ∼ treat, id=centre, weights = 1/nrep, family=gaussian(‘identity’), corstr=‘independence’)
	Cluster-level (unweighted)^b^	collapse (count) n = y (mean) y treat, by(centre) reg y treat	ybar <- as.numeric(tapply(y, centre, mean)) treatcl <- as.numeric(tapply(treat, centre, mean)) lm(ybar ∼ treatcl)

In the code columns, ‘y’ denotes a continuous participant-level outcome, ‘centre’ denotes cluster membership for each observation, ‘treat’ denotes intervention variable (0, 1) for each observation and ‘n’ denotes the number of participants included in each cluster.

a Weighted cluster-level analyses require the use of robust standard errors due to the use of the weights^46^ [these standard errors are not cluster-robust, as they are for independence estimating equations (IEEs) but are robust to heteroskedasticity]. The Stata option [pweight=n] implements this automatically, whereas R code requires loading package library(sandwich) and using the vcovHC(model, type=‘HC0’) option.

b The unweighted cluster-level analysis assumes that the marginal variance of each cluster mean is the same; if this is not the case, robust standard errors can be used to provide valid inference (though it may require a small-sample correction when the number of clusters is small).

## Unbiased estimators for the cluster-average treatment effect

As noted above, standard estimators used in CRTs (i.e. mixed-effects models with a random intercept and GEEs with an exchangeable working correlation structure) are also biased for the cluster-average treatment effect when cluster size is informative. We provide a summary of alternative estimators in Table 3.

## Example

Consider a trial comparing a quality improvement (QI) intervention to improve outcomes in patients undergoing emergency laparotomy.^37^ This intervention involves local QI leads implementing a hospital-wide improvement programme at each cluster. The primary outcome is overall mortality within 90 days and a secondary outcome is whether a senior surgeon is present in the operating theatre (either performing the surgery or supervising a more junior surgeon in doing so). This outcome is intended to measure the success of the QI intervention in changing hospital practice.

For the primary outcome, we need to decide whether a participant-average or cluster-average treatment effect is desired (i.e. do we want to know the average mortality reduction across patients or across hospitals?) Here, interest clearly lies in the intervention effect on individual patients (i.e. how many additional lives can be saved through the QI intervention?). Thus, a participant-average treatment effect is most relevant here.

However, the key secondary outcome (whether a senior surgeon is present) is intended to measure treatment success at the cluster level (i.e. whether the intervention was effective in making hospitals change their practice around emergency laparotomies). Hence, for this outcome, a cluster-average estimand may be the most relevant.

We note that for the secondary outcome (whether a senior surgeon is present), both a participant-average and cluster-average treatment effect may be of scientific interest, in which case both could be specified (e.g. with the cluster-average treatment effect designated as the primary^38^). However, including both estimands should only be done if both are indeed of scientific interest. For the primary outcome (overall mortality), it is unlikely the cluster-average treatment effect would be of scientific interest and so we would recommend only specifying the participant-average estimand for this outcome.

Once the estimand has been decided (including other aspects, such as population, handling of intercurrent events, etc.), we can decide on the estimator. We begin with the primary outcome (overall mortality), in which a participant-average treatment effect is of interest.

In this trial, it is plausible that success in implementing the QI intervention may differ between smaller and larger clusters due to differing resource levels available, resulting in an interaction between treatment effect and cluster size. It is also possible that mortality rates may differ between smaller and larger clusters due to differences between patients who present to each.

Thus, informative cluster size cannot be ruled out and so mixed-effects models or GEEs with an exchangeable working correlation structure should not be used. Instead, the primary outcome could be analyzed using IEEs. This could be implemented using GEE with a working independence correlation structure and robust standard errors, using the appropriate link function to provide the desired treatment effect (e.g. risk difference, risk ratio, odds ratio). Depending on the number of clusters in the trial, a small-sample correction may also be required to obtain valid standard errors. Alternatively, a cluster-level analysis weighted by the cluster size, with robust standard errors (see Table 3), could be used.

For the secondary outcome (presence of a senior surgeon), the most straightforward estimator is an analysis of unweighted cluster-level summaries (e.g. based on the proportion of cases in which a senior surgeon is present). This estimator is unbiased for the cluster-average treatment effect even in the presence of informative cluster sizes and has good statistical properties even when the number of clusters is small.

If desired, further analyses could explore variation in treatment effects across differently sized clusters. However, we note these analyses can be challenging to interpret, as it is unlikely to be the cluster size itself that modifies the treatment effect but rather some underlying factor associated with size (e.g. urban vs rural, differences in patient characteristics, staffing capacity or expertise in each cluster, etc.). Thus, it may be more relevant from a policy-making perspective to explore variation in treatment effects by these types of factors^39^^,^^40^ rather than by cluster size.

## Other implications

In principle, the difference in estimands described here (participant-average vs cluster-average) also applies to other cluster trial designs, such as cluster-crossover trials and stepped-wedge trials. However, for more complex longitudinal cluster-randomized designs, participant observations may be collected in different time periods, which may necessitate additional considerations in how to combine or average information across time. We defer those specific discussions to future work.

In practice, the choice of estimand should be made when designing the trial, as it is required for sample size estimation and to help plan the statistical analysis. It should be listed in the trial protocol and reported in the trial publication, to allow those using trial results to make informed decisions. To facilitate this, future revisions to the CONSORT and SPIRIT extension to CRTs^41^ (or stepped-wedge^42^ or cluster-crossover trials) should require this information.

The estimators considered here (Table 3) typically require a large number of clusters in order to provide valid standard errors. Therefore, when using these estimators with a small number of clusters, it is important to use a small-sample correction to ensure valid results.^24^^,^^43–45^ This is an active area of study and further research is required to identify the optimal approach for each estimator across various small-sample settings.

## Conclusion

Our concluding message is that though statistical issues such as bias in standard errors and inflated type I error rate are important, they should not be the driving force in the choice of statistical analysis of CRTs. Different statistical methods can fundamentally alter the question being addressed so researchers must define their target estimand at the outset to clarify the question they wish to address and carefully evaluate the potential for informative cluster size. Then, suitable methods of analysis can be chosen to address the right question while also maintaining statistical validity.

## Ethics approval

This article does not use real data sources and thus ethics approval is not relevant.

## Supplementary Material

dyac131_Supplementary_Data

## Data Availability

This article does not use any data, so there is no data to share.
