# Supplementary material for: Estimands in cluster-randomized trials: choosing analyses that answer the right question
Source: Int J Epidemiol. 2022 Jul 14;52(1):107–18. doi: 10.1093/ije/dyac131 (PMC9908044; doi:10.1093/ije/dyac131)
Supplement: dyac131_Supplementary_Data [file dyac131_supplementary_data.docx]

**Supplementary appendix: *Estimands in cluster-randomised trials: choosing analyses that answer the right question***

This appendix contains the Stata code used in the simulation study for figure 1.

**Stata code**

local n_rep = 2000

/* parameters */

local n_clusters = 60

local n_patients_small = 10

local n_patients_large = 100

local e_sd = 5

local beta_small = 5

local beta_large = 1

local alpha = 0

set seed 2344

set more off

version 16.1

/*** looping over ICC values ****/

foreach icc in 0.01 0.05 0.10 {

local u_sd = sqrt(`icc'*`e_sd'^2/(1-`icc'))

local new_icc = 100*`icc'

*** opening postfile ***/

cap postclose mysim

postfile mysim ///

///

estimand_partic_ave estimand_clust_ave ///

///

beta_ri beta_gee_exch beta_iee ///

using "N:\Documents\Simulated datasets\bias mixed models - icc eq `new_icc'", replace

*for loop

forval i = 1/`n_rep' {

if mod(`i', 5) == 0 {

dis `i'

}

qui {

**** calculating value of estimands ****

local estimand_partic_ave = ///

( (`n_clusters'/2)*(`n_patients_small')*`beta_small' + ///

(`n_clusters'/2)*(`n_patients_large')*`beta_large' ) / ///

( (`n_clusters'/2)*(`n_patients_small') + ///

(`n_clusters'/2)*(`n_patients_large') )

local estimand_clust_ave = ///

( (`n_clusters'/2)*`beta_small' + ///

(`n_clusters'/2)*`beta_large' ) / ///

( (`n_clusters'/2) + (`n_clusters'/2) )

/**** generating dataset ****/

clear

set obs `n_clusters'

gen id = _n // cluster id

*cluster level random effect

gen u_cluster = rnormal(0, `u_sd')

*trt allocations

egen z = seq(), from(0) to(1)

*number participants/cluster

gen cluster_large = 0 if id <= _N/2

replace cluster_large = 1 if id > _N/2

gen n_per_cluster = `n_patients_small' if cluster_large == 0

replace n_per_cluster = `n_patients_large' if cluster_large == 1

/*** reshaping/expanding ****/

expand n_per_cluster

*random error (patients)

gen u_patient = rnormal(0, `e_sd')

/* generating outcomes */

*outcome under no treatment

gen y = `alpha' + u_cluster + u_patient

replace y = y + `beta_small'*z if cluster_large == 0

replace y = y + `beta_large'*z if cluster_large == 1

/********* analysis *************

*

********************************/

*random-intercepts

mixed y z || id:,

matrix analysis_results = r(table)

local beta_ri = analysis_results[1,1] // treatment effect

*GEEs (exch corr)

xtset id

xtgee y z, family(normal) link(identity) corr(exch) vce(robust)

matrix analysis_results = r(table)

local beta_gee_exch = analysis_results[1,1] // treatment effect

*IEE

reg y z, vce(cluster id)

matrix analysis_results = r(table)

local beta_iee = analysis_results[1,1] // treatment effect

} // end quietly command

***** posting results to new dataset *****

*

******************************************

post mysim ///

///

(`estimand_partic_ave') (`estimand_clust_ave') ///

///

(`beta_ri') (`beta_gee_exch') (`beta_iee')

*closing for-loop

}

*closing postfile

postclose mysim

} // closing ICC loops

/**** getting results/generating graph *****

*

*******************************************/

cap postclose mysim2

postfile mysim2 ///

///

icc estimand_value mean_beta_ri mean_gee_exch mean_iee ///

///

using "N:\Documents\Simulated datasets\mix models bias for differeing icc values - results", replace

foreach n_icc in 1 5 10 {

use "N:\Documents\Simulated datasets\bias mixed models - icc eq `n_icc'", clear

summ estimand_partic_ave

local estimand_value = r(mean)

summ beta_ri

local mean_beta_ri = r(mean)

summ beta_gee_exch

local mean_gee_exch = r(mean)

summ beta_iee

local mean_iee = r(mean)

post mysim2 ///

///

(`n_icc') (`estimand_value') (`mean_beta_ri') (`mean_gee_exch') (`mean_iee')

*closing for-loop

}

*closing postfile

postclose mysim2

/**** figure *****

*

*****************/

use "N:\Documents\ \Simulated datasets\mix models bias for differeing icc values - results", clear

foreach i in beta_ri gee_exch iee {

gen percent_bias_`i' = 100*(mean_`i'-estimand_value)/estimand_value

}

set scheme s1color

twoway ///

(scatter percent_bias_beta_ri icc, mcolor("220 36 31") msymbol(Oh) connect(direct) lcolor("220 36 31")) || ///

(scatter percent_bias_gee_exch icc, mcolor("0 25 168") msymbol(Dh) connect(direct) lcolor("0 25 168")) ///

(scatter percent_bias_iee icc, mcolor("0 114 41") msymbol(Th) connect(direct) lcolor("0 114 41")), ///

///

yline(0, lcolor(black) lpattern(dot)) ///

xtitle("ICC") xlabel(1 "0.01" 5 "0.05" 10 "0.10") ///

ytitle("Percent bias") ylabel(-20 0 20 40 60 80 100 120) ///

legend(label(1 "Mixed-effects model") label(2 "GEEs (exch corr)") label(3 "IEEs") ///

ring(0) bplacement(nwest) col(1))
